# Seasonality and transmissibility of *Plasmodium ovale* in Bagamoyo District, Tanzania

**DOI:** 10.1186/s13071-022-05181-2

**Published:** 2022-02-14

**Authors:** Brian B. Tarimo, Vincent O. Nyasembe, Billy Ngasala, Christopher Basham, Isaack J. Rutagi, Meredith Muller, Srijana B. Chhetri, Rebecca Rubinstein, Jonathan J. Juliano, Mwajabu Loya, Rhoel R. Dinglasan, Jessica T. Lin, Derrick K. Mathias

**Affiliations:** 1 Vector Immunity and Transmission Biology Unit, Department of Environmental Health and Ecological Science, Ifakara Health Institute-Bagamoyo Office, P.O. Box 74, Bagamoyo, Coast Region 61301 Tanzania; 2 Department of Infectious Diseases and Immunology, College of Veterinary Medicine, Emerging Pathogens Institute, University of Florida, Gainesville, FL 32611 USA; 3 Department of Parasitology and Medical Entomology, School of Public Health and Social Sciences, Muhimbili University of Health and Allied Sciences, Dar es Salaam, 11103 Tanzania; 4 Institute of Global Health and Infectious Diseases, University of North Carolina School of Medicine, Chapel Hill, NC 27599 USA; 5 Department of Entomology and Nematology, Florida Medical Entomology Laboratory, Institute of Food and Agricultural Sciences, University of Florida, Vero Beach, FL 32962 USA

**Keywords:** *Plasmodium ovale*, *Plasmodium falciparum*, Malaria, Asymptomatic, Tanzania, *Anopheles gambiae*, Direct feeding assay (DFA)

## Abstract

**Background:**

*Plasmodium ovale* is a neglected malarial parasite that can form latent hypnozoites in the human liver. Over the last decade, molecular surveillance studies of non-falciparum malaria in Africa have highlighted that *P. ovale* is circulating below the radar, including areas where *Plasmodium falciparum* is in decline. To eliminate malaria where *P. ovale* is endemic, a better understanding of its epidemiology, asymptomatic carriage, and transmission biology is needed.

**Methods:**

We performed a pilot study on *P. ovale* transmission as part of an ongoing study of human-to-mosquito transmission of *P. falciparum* from asymptomatic carriers. To characterize the malaria asymptomatic reservoir, cross-sectional qPCR surveys were conducted in Bagamoyo, Tanzania, over three transmission seasons. Positive individuals were enrolled in transmission studies of *P. falciparum* using direct skin feeding assays (DFAs) with *Anopheles gambiae* s.s. (IFAKARA strain) mosquitoes. For a subset of participants who screened positive for *P. ovale* on the day of DFA, we incubated blood-fed mosquitoes for 14 days to assess sporozoite development.

**Results:**

Molecular surveillance of asymptomatic individuals revealed a *P. ovale* prevalence of 11% (300/2718), compared to 29% (780/2718) for *P. falciparum*. Prevalence for *P. ovale* was highest at the beginning of the long rainy season (15.5%, 128/826) in contrast to *P. falciparum*, which peaked later in both the long and short rainy seasons. Considering that these early-season *P. ovale* infections were low-density mono-infections (127/128), we speculate many were due to hypnozoite-induced relapse. Six of eight *P. ovale*-infected asymptomatic individuals who underwent DFAs successfully transmitted *P. ovale* parasites to *A. gambiae*.

**Conclusions:**

*Plasmodium ovale* is circulating at 4–15% prevalence among asymptomatic individuals in coastal Tanzania, largely invisible to field diagnostics. A different seasonal peak from co-endemic *P. falciparum*, the capacity to relapse, and efficient transmission to *Anopheles* vectors likely contribute to its persistence amid control efforts focused on *P. falciparum*.

**Graphical Abstract:**

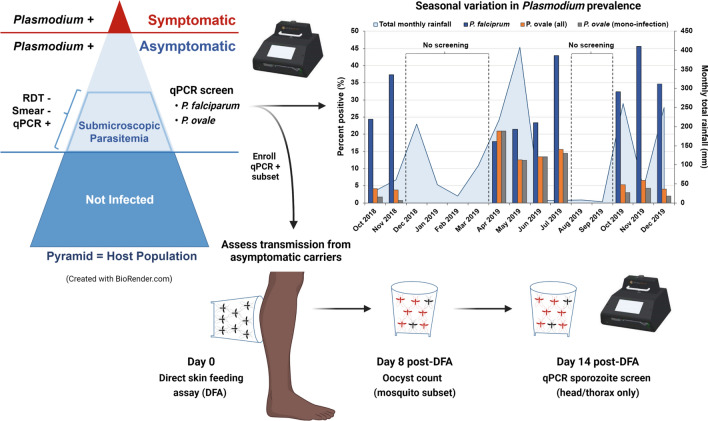

Molecular surveillance studies of non-falciparum malaria in Africa over the last decade have highlighted that *Plasmodium ovale* is circulating below the radar [1, 2] and consists of two distinct species, *P. ovale curtisi* and *P. ovale wallikeri* [3, 4], that are rising in prevalence, including in areas where *Plasmodium falciparum* is in decline [5–8]. These studies have been primarily country-level surveys conducted intermittently over several years, while fewer studies have examined the local epidemiology driving these trends. We applied *P. ovale*-specific real-time polymerase chain reaction (qPCR) assays in the context of an ongoing study of the asymptomatic *P. falciparum* infectious reservoir in Bagamoyo, Tanzania, to examine the seasonality and transmissibility of *P. ovale* sensu lato (hereafter *P. ovale*). Herein, we describe the results of cross-sectional surveys across three malaria transmission seasons and of a pilot study that used direct skin feeding assays (DFAs) in subjects with *P. falciparum/P. ovale* infection to explore their infectivity to mosquitoes.

Bagamoyo district in coastal Tanzania has moderate malaria transmission that occurs throughout the year, with peaks during the long (March to June) and short (October to December) rainy seasons. *Anopheles gambiae* sensu stricto, *Anopheles arabiensis* and *Anopheles funestus* s.s. are the main vectors [9]. Participants aged 6 years and above without fevers or chills in the preceding 3 days were recruited from schools and health centers in the villages of Fukayosi, Mwavi, and Yombo for screening during the long and short rainy seasons from October 2018–December 2019, as well as for 1 month into the dry season following the long rains in 2019.

Finger-prick blood was used to make thick and thin blood smears, perform dual-antigen histidine-rich protein 2 (HRP2) and parasite lactate dehydrogenase (pLDH) rapid diagnostic tests (RDT; SD Bioline), and create two to three dried 50 µl blood spots (DBS) on Whatman 3MM filter paper. Species-specific *P. falciparum* qPCR targeting 18S rRNA was performed on DBS-extracted DNA as described [10]. For *P. ovale*, primers and probe targeting an 18S rRNA region conserved in *P. ovale curtisi* and *P. ovale wallikeri* were used [2]. Midway through the study, qPCR was shifted to Bagamoyo, with the same protocol modified for use with Sahara Hot Start PCR Master Mix (Chai Biotechnologies). For *P. falciparum* detection, samples were run in duplicate and considered positive if the cycle threshold (*C*_t_) was < 40 cycles in at least one reaction. For *P. ovale* detection, samples were initially run singly and considered positive if *C*_t_ was < 40 cycles. Since low-density parasitemia is common in *P. ovale* infections, samples were also considered positive if *C*_t_ ≥ 40 but < 45 and then confirmed by qPCR in a subsequent independent reaction. All participants positive for malaria by microscopy and/or RDT were treated with artemisinin combination therapy in accordance with Tanzanian national guidelines.

Female *A. gambiae* s.s. (IFAKARA strain) mosquitoes (4–7 days post-emergence) were used in all mosquito feeding assays. The IFAKARA colony was established in 1996 from mosquitoes collected in southeastern Tanzania [11] and has been maintained in the laboratory since then. To generate experimental mosquitoes, eggs were collected on moist filter paper, rinsed with 1% bleach, and hatched in plastic pans containing deionized water. Larvae were reared in plastic bowls in deionized water and fed daily with TetraMin^®^ fish food. Pupae were transferred to 30 × 30 × 30 cm cages in a climate-controlled room at a constant temperature of 27 °C and relative humidity of 70–80%. Emerged adults were maintained in the same cages on 10% sucrose solution until they were transferred to 500 ml mesh-covered cups for feeding assays. Mosquitoes not used in experiments were fed blood (acquired from a blood bank through Bagamoyo District Hospital) using a Hemotek artificial feeding system to produce eggs for colony maintenance.

Participants positive by RDT or *P. falciparum* qPCR were enrolled for mosquito direct skin feeding assays (DFAs) on the same day (RDT+) or within 6 days of screening (PCR+). For a subset who were qPCR-positive for *P. ovale* on the day of DFA, mosquito-dissection procedures were modified to enable molecular evaluation for sporozoite development. Briefly, female mosquitoes were prepared as described above and starved 4–6 h prior to DFAs. For each assay, a cup with 25 mosquitoes was attached to each calf of a participant (50 mosquitoes/participant) and allowed to feed for 15 min. If the participant expressed discomfort, the DFA was stopped immediately. Blood-fed mosquitoes were kept in climate-controlled incubators (27 °C, 70–80% humidity, 12:12 h light/dark) for 24 h without access to sucrose or water to kill unfed mosquitoes and were maintained on 10% sucrose thereafter for the duration of the experiment. On day 8 post-DFA, midguts from a subset of mosquitoes were dissected, stained with 0.1% Mercurochrome, and scored for oocysts. Positive midguts were individually stored in DNA/RNA Shield (Zymo Research) for *Plasmodium* screening by qPCR. If one or more oocyst-positive midguts were found in the first 10 dissected, the remaining mosquitoes were maintained until day 14 post-DFA, when mosquito abdomens were removed and heads/thoraxes were pooled in groups of up to five for DNA extraction and qPCR.

DNA from mosquito heads/thoraxes stored in DNA/RNA Shield was extracted using the Quick-DNA Tissue/Insect Kit (Zymo Research) following the manufacturer’s instructions. DNA from oocyst-positive midguts stored in DNA/RNA Shield was extracted using DNAzol Reagent (Invitrogen). Prior to homogenization, samples were centrifuged at 3000×*g* for 15 s, DNA/RNA Shield was removed, and 100 µl of DNAzol was added. Samples were homogenized with a sterilized pestle, brought to 1 ml with DNAzol, and incubated at room temperature for 2 min. After incubation, 0.2 µg of glycogen and 500 µl of −20 °C ethanol was added, and samples were centrifuged at 12,000×*g* for 10 min at 4 °C. The remaining steps followed the DNAzol manufacturer’s instructions. Species-specific qPCR was performed as described above.

Malaria screening and mosquito-infection data were entered into a RedCAP database. The prevalence of *P. falciparum* and *P. ovale* was calculated for each enrollment period. Associations with sex, age, and season were analyzed by log-binomial regression in Stata v.16.1. Rainfall data for the study area were acquired from the Tanzania Meteorological Agency (TMA; https://www.meteo.go.tz). The transmissibility of *P. ovale* parasite carriers was assessed based on frequency of mosquito midgut and salivary gland/sporozoite infection.

Over three transmission seasons (October 2018 to December 2019), 2718 asymptomatic individuals aged 6 years and older underwent malaria screening. Overall, *P. falciparum* prevalence by qPCR was 29% (780/2718), while 15% (382/2718) were positive by RDT (HRP2 band), and 12% (316/2718) were smear-positive. *Plasmodium ovale* prevalence by qPCR was 11% (300/2718), ranging from 4 to 15% (Table 1). Only 7.7% (23/300) of *P. ovale* qPCR-positive infections had any parasites (*P. falciparum* or *P. ovale*) detected by microscopy, while 9.7% (29/300) were diagnosed with malaria based on a positive RDT (HRP2 band present indicating presence of *P. falciparum* antigen). Thus, approximately 90% (271/300) of asymptomatic *P. ovale* infections were invisible to routine malaria diagnostic testing, compared to roughly half (54%, 422/780) of asymptomatic *P. falciparum* infections.

**Table 1:** Characteristics of participants screened for *Plasmodium* during the study period

Characteristics	Total	*P. ovale* status^a^	
		Positive	Negative
Number screened	2718	300 (11%)	2418 (89%)
Age, median (IQR)	20 (11–32)	23 (12–32)	19 (11–32)
Children/adolescents (≤ 17 years old)	1222 (45%)	112 (9.2%)	1110 (91%)
Adults (≥ 18 years old)	1496 (55%)	188 (13%)	1308 (87%)
Sex			
Female	1831 (67%)	202 (11%)^b^	1629
Male	887 (33%)	98 (11%)	789
*P. falciparum*			
Positive^a^	780 (29%)	33 (11%)^c^	747
Negative	1938 (71%)	267 (89%)^d^	1671
RDT			
Positive	382 (14%)	29 (10%)^b^	353
Negative	2336 (86%)	271 (90%)	2065
Blood smear			
Positive	316 (12%)	23 (7.7%)^b^	293
Negative	2402 (88%)	277 (92%)	2125
Season			
Oct–Nov 2018 (short rains)	527 (19%)	21 (4.0%)^e^	506
Apr–May 2019 (long rains, early)	826 (30%)	128 (15%)	698
Jun–Jul 2019 (long rains, late)	829 (31%)	120 (14%)	709
Oct–Dec 2019 (short rains)	536 (20%)	31 (5.8%)	505

Data are shown for all individuals screened and by *P. ovale* infection status

^a^*Plasmodium ovale* and *P. falciparum* status were determined by 18S qPCR; subjects deemed positive if the *C*_t_ value of the first qPCR was < 40 or if the mean *C*_t_ value of the first qPCR and at least one follow-up qPCR was < 45

^b^Percentages in this column for sex, RDT, and blood smear are from the total who were qPCR-positive for *P. ovale*

^c^Percentage of *P. ovale*-positive individuals co-infected with *P. falciparum*

^d^Percentage of *P. ovale*-positive individuals with mono-infections

^e^Percentages in the *P. ovale*-positive column indicate season-specific prevalence

Parasite prevalence varied significantly by season with *P. falciparum* and *P. ovale* infections peaking at different times of the year (Fig. 1). While *P. falciparum* prevalence was highest during the short rainy seasons in October–November (32% and 39% for both months combined, respectively, in 2018 and 2019), *P. ovale* prevalence peaked early in the long rainy season in April–May 2019 (16%), reaching comparable numbers to *P. falciparum* in April (21% vs. 18% for *P. ovale* and *P. falciparum,* respectively). *Plasmodium ovale* prevalence subsequently dropped three- to fourfold during the October–November short rainy season when *P. falciparum* cases were highest.

**Fig. 1:**
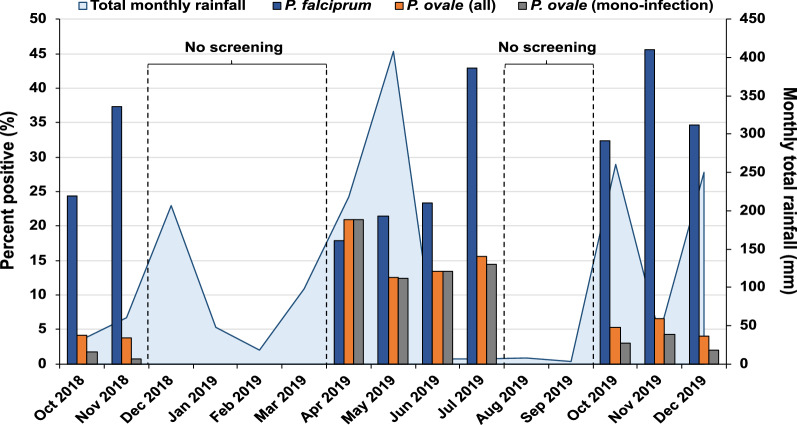
Monthly parasite prevalence (left *Y*-axis) among asymptomatic participants for *P. falciparum* (blue bars), *P. ovale* (orange bars), and *P. ovale* mono-infections (gray bars). The total monthly rainfall is denoted by the blue line (right *Y*-axis)

*Plasmodium falciparum*/*P. ovale* co-infections were not common overall (33 total, or 4.2% of *P. falciparum* [33/780] and 11% of *P. ovale* [33/300] infections) (Table 1), but they exhibited striking seasonality (Fig. 1). The majority of *P. falciparum*/*P. ovale* co-infections occurred during the short rainy season in October–November, when 71.4% (15/21) and 38.7% (12/31) of *P. ovale* infections were mixed in 2018 and 2019, respectively. In contrast, all but one of the 128 *P. ovale* infections detected during April–May 2019 were mono-infections. These had higher qPCR *C*_*t*_ values on average compared to *P. ovale* infections detected in all other seasons combined (*C*_*t*_ = 41.0 vs. 39.8, *t*_(298)_ = 3.79, *P* < 0.001), suggesting lower parasite densities.

Male sex and younger age were associated with increased risk of infection with *P. falciparum* but not *P. ovale*. For *P. falciparum,* males were 1.5 times more likely to be infected compared to females (RR = 1.5, *P* < 0.001), and the risk of infection decreased slightly with each year in age (RR = 0.99, *P* < 0.001). In contrast, sex and age were not associated with *P. ovale* infection risk when examined as continuous variables, either in univariate analysis or when the model included sex, age, and season. However, the median age of *P. ovale*-positive versus negative persons was greater by 4 years (Table 1), and the point prevalence of *P. ovale* was slightly higher in adults aged 18 years and older (12.6%, 188/1496) compared to children and adolescents 6–17 years of age (9.2%, 112/1222) (*Z* = −2.82, *P* = 0.005). For *P. falciparum/P. ovale* co-infections, each yearly increase in age reduced the relative risk of co-infection by 4.3% (RR = 0.96, *P* = 0.04), with males being more likely to be co-infected than females (RR = 3.3, *P* = 0.001).

Over 4 weeks in October–November 2019, qPCR screening of participants undergoing DFA detected *P. ovale* in eight participants, aged 9–52 years (median 23.5 years). Mosquitoes fed on these participants who harbored *P. ovale* on the day of DFA were evaluated for *P. falciparum* and *P. ovale* infection. Five participants were PCR-positive for mixed *P. falciparum/P. ovale* infection, while three had *P. ovale* mono-infection by qPCR (Table 2). The majority of DFAs (6 of 8) yielded *P. ovale* infections in mosquitoes at either day 8 or day 14 post-DFA, regardless of the mixed infection status observed at screening or enrollment (Table 2). In all four experiments where pooled mosquitoes (head and thoraxes) were available for testing at day 14 post-DFA, the presence of *P. ovale* sporozoites was detected by qPCR, with all but one experiment yielding mosquitoes infected solely or predominantly with *P. ovale*.

**Table 2:** Results of human-to-mosquito transmission experiments from subjects recruited after screening who were positive for *P. ovale* infection by PCR on day of DFA (direct feeding assay)

Subject ID	Blood PCR^*a*^ on day of DFA		No. of positive midguts	Midgut PCR at D8 post-feeding		No. of PCR+ mosquito pools at D14 post-feeding		*P. ovale*-positive mosquitoes only^*b*^	*P. falciparum*- & *P. ovale*-positive mosquitoes
	*P. falciparum*	*P. ovale*		*P. falciparum*	*P. ovale*	*P. falciparum*	*P. ovale*		
**MqTZ-2312**	++	++	1/3	ND	ND	0/7	6/7	✓	✗
**MqTZ-2390**	+++	+	3/10	++	+	5/6	4/6	✗	✓
MqTZ-2394	+++	+++	3/47	–	+	ND	ND	✓	✗
**MqTZ-2396**	+++	++	1/10	++	+/–	0/6	5/6	✓	✗
MqTZ-2431	–	+++	3/48	–	++	ND	ND	✓	✗
**MqTZ-2499**	+++	+++	5/10	++	+	1/14	8/14	✗	✓
MqTZ-2556	–	+	1/34	+	++	ND	ND	✗	✗
MqTZ-2563	–	+++	0/43	ND	ND	ND	ND	✗	✗

In all DFA experiments, mosquito midguts were dissected at day 8 (D8) post-feeding for detection of parasite oocysts by microscopy. In four subjects (subject ID in boldface), additional mosquitoes were held until day 14 (D14) post-feeding to assess sporozoite development by qPCR

^a^For qPCR results, +++ denotes a mean *C*_*t*_ value ≤ 32, ++ denotes a mean *C*_*t*_ value > 32 but < 40, + denotes a mean *C*_*t*_ value ≥ 40, and – indicates that no qPCR product was detected. For qPCRs with a *C*_*t*_  ≥ 40, only those with a confirmatory follow-up qPCR are reported

^b^*Plasmodium ovale* infection status determined at D14

We report the comparative prevalence of *P. falciparum* and *P. ovale* among asymptomatic carriers in coastal Tanzania over a 14-month period and demonstrate human-to-mosquito transmission of *P. ovale* parasites to *A. gambiae* s.s. mosquitoes. *Plasmodium ovale* prevalence was substantial, ranging from 4 to 15%, with 90% of this burden invisible to routine diagnostics (RDT and microscopy).

Our longitudinal analysis reveals a seasonality to *P. ovale* cases that contrasts with co-endemic *P. falciparum*, peaking near the beginning of the long rainy season in April, when *P. falciparum* prevalence is relatively lower. This seasonal pattern could be due to variability in transmission of the two parasite species driven by seasonal differences in *Anopheles* spp. abundance combined with varying transmission efficiency for certain vector–parasite combinations. Nothing is known about which anopheline species efficiently transmit *P. ovale*, but our pilot study suggests that *P. ovale* readily infects *A. gambiae*, historically the main vector for *P. falciparum* in the region. These observations, along with the relatively high proportion of *P. falciparum/P. ovale* co-infections in October–November and reported in other studies [2, 5, 6], suggest that the two parasite species are frequently co-transmitted in the same mosquitoes, a phenomenon we also observed in two DFA participants.

Notably, the early-season *P. ovale* cases in April–May were virtually all mono-infections and exhibited lower parasite densities than co-infections occurring in October–November. This raises the question of whether the influx of low-density mono-infections during these months arose from hypnozoite-induced relapse [12]. Seasonal patterns of *P. vivax* versus *P. falciparum* have previously been used to infer the proportion of vivax cases due to relapse [13, 14]. While there is still much to be learned about the relapse patterns of *P. ovale*, relapses occurring in April–June, 4 to 6 months following mosquito bites in October–November, could be consistent with the relapse intervals (a median of 15 weeks) observed in a longitudinal cohort study of *P. ovale*-infected persons in Gabon [15]. Interestingly, the seasonal pattern of 77 imported cases of *P. ovale* in China from 2010 to 2017 resembles our observations, with cases peaking slightly higher in April than October [16]. We speculate that relapses may have evolved to occur when anopheline vectors are increasing in abundance at the onset of the rainy season.

The use of skin feeding assays combined with on-site PCR detection allowed us to highlight what appears to be highly efficient human-to-mosquito transmission of *P. ovale* from asymptomatic carriers invisible to routine diagnostics, including those with mixed-species infection. There is much more to be learned about whether this holds true during different seasons, with different anopheline vectors, and for different *P. ovale* species (*P. ovale curtisi* vs. *P. ovale wallikeri*).

Limitations to this study include its cross-sectional nature, small sample of subjects in the DFA study, lack of data in the months between rainy seasons, and the use of insectary-reared mosquitoes to assess infectivity. We have not yet determined the species of these *P. ovale* isolates (as *P. ovale curtisi* or *P. ovale wallikeri*), many of which are low-density samples.

We provide the first direct evidence of efficient transmission from asymptomatic *P. ovale* carriers to *A. gambiae* and seasonal patterns of *P. ovale* infection in coastal Tanzania that may provide clues to how this species, which remains largely undetected, continues to circulate amid heightened efforts to control and eliminate malaria.

## Data Availability

The datasets used and/or analyzed during the current study are available from the corresponding author upon request.

## Abbreviations

*A. gambiae*: *Anopheles gambiae*
*C*_t_: Cycle threshold
DBS: Dried blood spot
DFA: Direct skin feeding assay
IQR: Interquartile range
*P. falciparum*: *Plasmodium falciparum*
*P. ovale*: *Plasmodium ovale*
qPCR: Quantitative polymerase chain reaction
RDT: Rapid diagnostic test
RR: Risk ratio
rRNA: Ribosomal RNA
